# The Impact of Low-dose Gliclazide on the Incretin Effect and Indices of Beta-cell Function

**DOI:** 10.1210/clinem/dgab151

**Published:** 2021-03-08

**Authors:** Ruth L M Cordiner, Andrea Mari, Andrea Tura, Ewan R Pearson

**Affiliations:** 1 Division of Population Health and Genomics, School of Medicine, University of Dundee, UK; 2 Institute of Neuroscience, National Research Council, Padua, Italy

**Keywords:** sulphonylureas, gliclazide, incretins, incretin effect, beta-cell physiology, type 2 diabetes, KATP channel, GLP-1, GIP, beta-cell modeling

## Abstract

**Aims/Hypothesis:**

Studies in permanent neonatal diabetes suggest that sulphonylureas lower blood glucose without causing hypoglycemia, in part by augmenting the incretin effect. This mechanism has not previously been attributed to sulphonylureas in patients with type 2 diabetes (T2DM). We therefore aimed to evaluate the impact of low-dose gliclazide on beta-cell function and incretin action in patients with T2DM.

**Methods:**

Paired oral glucose tolerance tests and isoglycemic infusions were performed to evaluate the difference in the classical incretin effect in the presence and absence of low-dose gliclazide in 16 subjects with T2DM (hemoglobin A1c < 64 mmol/mol, 8.0%) treated with diet or metformin monotherapy. Beta-cell function modeling was undertaken to describe the relationship between insulin secretion and glucose concentration.

**Results:**

A single dose of 20 mg gliclazide reduced mean glucose during the oral glucose tolerance test from 12.01 ± 0.56 to 10.82 ± 0.5mmol/l [*P* = 0.0006; mean ± standard error of the mean (SEM)]. The classical incretin effect was augmented by 20 mg gliclazide, from 35.5% (lower quartile 27.3, upper quartile 61.2) to 54.99% (34.8, 72.8; *P* = 0.049). Gliclazide increased beta-cell glucose sensitivity by 46% [control 22.61 ± 3.94, gliclazide 33.11 ± 7.83 (*P* = 0.01)] as well as late-phase incretin potentiation [control 0.92 ± 0.05, gliclazide 1.285 ± 0.14 (*P* = 0.038)].

**Conclusions/Interpretation:**

Low-dose gliclazide reduces plasma glucose in response to oral glucose load, with concomitant augmentation of the classical incretin effect. Beta-cell modeling shows that low plasma concentrations of gliclazide potentiate late-phase insulin secretion and increase glucose sensitivity by 50%. Further studies are merited to explore whether low-dose gliclazide, by enhancing incretin action, could effectively lower blood glucose without risk of hypoglycemia.

The incretin effect is defined as amplification of insulin secretion following oral glucose as opposed to the same stimulus given intravenously to provide identical plasma glucose concentrations; this is due to the postprandial insulin response being mediated by the release of incretin hormones from the gastrointestinal tract (1). The incretin effect is diminished or absent in type 2 diabetes mellitus (1), related to the diminished insulinotropic effect of glucose‐dependent insulinotropic polypeptide (GIP) (2,3). The loss of the incretin effect is targeted therapeutically by inhibiting breakdown of GIP and glucagon‐like peptide 1 (GLP-1) via dipeptidyl-peptidase 4 or by use of supraphysiological doses of GLP-1 receptor agonists.

Insulin secretion from the pancreatic beta-cell is controlled by 2 interacting pathways: the triggering pathway and the amplifying pathway (4), with the amplifying pathway requiring the triggering pathway to be effective. Sulphonylureas act by stimulating glucose-independent insulin secretion via closure of adenosine 5′-triphosphate-sensitive potassium (K_ATP_) channels (5), thereby activating the triggering pathway. In contrast, incretins and drugs augmenting the incretin pathway activate the amplifying pathway, resulting in glucose-dependent insulin secretion. A key defect in type 2 diabetes lies in the amplifying pathway (6); however, the role of the triggering pathway in the defective incretin effect seen in type 2 diabetes is uncertain.

Patients with neonatal diabetes due to activating mutations in the genes encoding the K_atp_ channel provide potential insight into the role of the triggering pathway and potential interaction with incretin action (7-10). Patients with these forms of neonatal diabetes can effectively transition off insulin onto high-dose sulphonylurea, and most achieve normoglycemia with no hypoglycemia, consistent with glucose-dependent insulin secretion (11). In physiological studies on these patients before and after use of sulphonylureas, there was only minimal increase in insulin secretion by sulphonylureas following intravenous glucose, yet a robust insulin secretory response was seen with an oral glucose stimulus consistent with sulphonylureas augmenting the incretin effect (11). Mechanistically, this can be explained as follows: in the absence of sulphonylureas, the activating mutations in the K_ATP_ channel result in the channels being insensitive to changes in intracellular adenosine 5′-triphosphate:adenosine 5′-diphospate, and so the beta-cell membrane remains hyperpolarized and the beta-cells are unable to secrete insulin (ie, the triggering pathway is blocked); with high-dose sulphonylurea treatment, there is closure of some of the K_ATP_ channels, sufficient to enable incretins to augment secretion but with only minimal activation of the triggering pathway. In parallels with neonatal diabetes, alteration in K_atp_ channel function also plays an etiological role in the development of type 2 diabetes with the E23K/S1119 KCNJ11/ABCC8 variants associated with a 1.32 increase in the risk of type 2 diabetes (12). Thus, similar, if less extreme, mechanisms may be seen in patients with type 2 diabetes.

We hypothesized that, like patients with neonatal diabetes, sulphonylureas might be able to promote incretin action with little or no direct effect on insulin secretion if used at a low enough dose in type 2 diabetes. Therefore, we have evaluated the incretin effect using the gold standard protocol of paired frequently sampled oral glucose tolerance tests (OGTT) and matched isoglycemic intravenous glucose infusions (IIGI) in patients with type 2 diabetes treated with acute low-dose sulphonylurea, a condition that does not stimulate insulin secretion markedly.

## Materials and Methods

### Recruitment

We utilized the Scottish Diabetes Research Network and SHARE network permissions to recruit 20 participants with type 2 diabetes mellitus controlled with no treatment or metformin monotherapy with hemoglobin A1c (HbA1c) < 64 mmol/mol (8.0%) aged between 40 and 80 years with estimated glomerular filtration rate ≥ 50 mL/min^−1^ and alanine aminotransferase ≤ 2.5 times the upper limit of normal. The study recruited only white British participants to avoid heterogeneity within the study. Exclusion criteria included inability to consent, type 1 diabetes mellitus, anemia (hemoglobin < 12.0 g/dL for women, <13.0 g/dL for men), significant renal or hepatic impairment, pregnancy, lactation or female planning to conceive within the study period, established pancreatic disease, or any other significant reason as determined by the investigators.

### Study design

Research involved a single site, parallel, proof-of-concept physiological study at the Clinical Research Centre at Ninewells Hospital (Dundee, UK). Ethical approval was obtained from the East of Scotland Research Ethics Committee (18/ES/0064) and the study was registered with ClinicalTrials.gov (NCT03705195). All research was conducted in accordance with the Declaration of Helsinki, and written informed consent was obtained for all participants prior to study inclusion. Participants were informed of the purpose of study as part of informed consent. Participants attended the research center for 5 separate visits.

An initial screening visit assessed eligibility and obtained informed written consent for study. In addition, a full medical history and examination were performed. Baseline vital signs, anthropometry, full blood count, urea and electrolytes, and baseline HbA1c were completed to confirm safety and eligibility.

**Figure 1.**
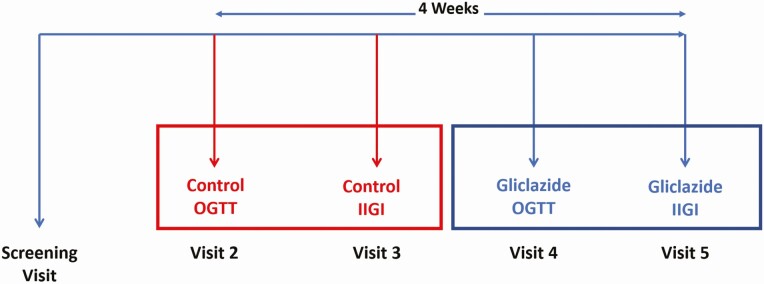
Study design comprising paired OGTT and IIGI in the presence (blue) and absence (red) of low-dose gliclazide.

The study design (Fig. 1) comprised a 4-h frequently sampled 75-g OGTT visit, followed on a separate day by a 4-h IIGI to mimic the plasma glucose curve obtained during the OGTT by means of an adjustable intravenous 20% glucose infusion. In the first 2 intervention visits (visits 2 and 3), a control OGTT, followed on a separate day by a control IIGI, were performed. At the next 2 visits (visits 4 and 5), oral liquid low-dose gliclazide was administered 1 h before an OGTT and, on a separate day, an IIGI. Study visits took place 1 week apart over a period of 4 weeks. Participants on prior metformin monotherapy omitted their dose on the morning of each visit to avoid postdose peak concentrations occurring during intervention.

### Study phases

To identify the most effective dose of gliclazide, the study was conducted in 2 phases. An exploratory first, dose-ranging phase (n = 8) was conducted that randomly allocated participants to receive either 10 mg (n = 4) or 20 mg (n = 4) of gliclazide. Phase 1 was unblinded but randomized in accordance with General Medical Council Good Clinical Practice utilizing computer-generated randomization conducted by the principal investigator (Research Randomizer, http://www.randomizer.org). Interim analysis was conducted following phase 1 to identify whether a 10-mg or 20-mg dose of gliclazide was more effective. The dose with greater effect was used in further (n = 12) participants in phase 2.

### Oral glucose tolerance test

Total test time was 240 min; liquid gliclazide was given 1 h prior to gliclazide intervention visits. Participants attended the Clinical Research Centre following a 10-h fast. A single intravenous cannula was sited in the participant’s antecubital fossa for blood collection. At time 0, participants consumed a drink containing 75 g of anhydrous glucose (133 mL Nutricia Polycal Liquid, Nutricia Limited, UK) diluted to 300 mL with water over a maximum 5-min period.

To enable glucose matching, bedside blood glucose level was analyzed at 5-min intervals in the first hour of study, 10 min in the second hour, and every 15 min in the final 2 h (Biosen GP+, EKF Diagnostics, Cardiff, UK). Blood samples were drawn at defined time-points for insulin, C-peptide, glucagon, glucose: 0, 5, 10, 15, 20, 30, 40, 45, 50, 60, 70, 90, 120, 150, 180, and 240 min. Total GLP-1 and GIP were measured over the same time course but during the OGTT only. In the intervention visits, gliclazide suspension (80 mg/5 mL gliclazide suspension, Alliance Pharmaceuticals, UK) was administered 60 min prior to oral glucose administration. Gliclazide concentration was sampled at 30-min intervals throughout visits 4 and 5.

### Isoglycemic intravenous glucose infusion

The IIGI protocol was obtained from the Steno Diabetes Center at Gentofte Hospital, Copenhagen, Denmark. An adjustable infusion of 20% glucose (Fresenius Kabi Ltd) was infused via infusion pump (Infusomat Space, B. Braun Medical, Sheffield, UK) according to bedside measurements of plasma glucose (13). Blood sampling and bedside measurements were performed in identical time course to OGTT. Further bedside sampling was occasionally required to maintain isoglycemia.

### Bedside glucose analysis

Bedside glucose analysis was performed utilizing the Biosen C-line GP+ (EKF Diagnostics, Cardiff, UK). Whole venous blood was taken up with a 20 µL end-to-end capillary, hemolyzed, and diluted in microtest tube, prefilled with a hemolyzing solution and then measured by the device via enzymatic-amperometry and chip-sensor technology. The system was set to correct and calibrate for use of whole-blood sampling. The accuracy of the isoglycemic clamp was confirmed after each experimental visit with formal blood glucose analysis.

### Gliclazide suspension

Gliclazide 80 mg/5 mL suspension was sourced through Alliance Pharmaceuticals. Respective 10-mg or 20-mg doses were administered orally via microdosing syringe.

### Blood collection

All blood collection was performed utilizing BD Vacutainer systems. Samples for insulin, C-peptide, glucagon, and gliclazide analysis were collected for EDTA plasma. Samples for glucose were collected in fluoride oxalate and collection for incretin analysis were obtained utilizing P800 blood collection system containing K_2_EDTA and protease inhibitors. Samples were iced following collection and centrifuged immediately in accordance with recommended guidance from receiving laboratories.

### Glucose solution for oral glucose tolerance test

Glucose solution for OGTT was prepared utilizing Nutricia Polycal liquid. One hundred thirteen milliliters of Polycal Liquid is equivalent to 75 g of anhydrous glucose, which was made up to a total volume of 300 mL with water as per standard NHS Tayside OGTT protocol.

### Laboratory analyses

#### Insulin and C-peptide

Analysis of insulin and C-peptide was performed by Clinical Chemistry, Royal Devon and Exeter Hospital—602 modules Cobas 8000 automated platform using sandwich chemiluminescence immunoassay (Elecsys insulin).

#### Glucose

Glucose analysis was performed by NHS Tayside Blood Sciences at Ninewells Hospital utilizing Siemens ADVIA Chemistry, Glucose Hexokinase_3 Concentrated Reagents.

#### Glucagon

Glucagon analysis was performed by the Immunoassay Core Biomarker Laboratory, University of Dundee, utilizing EMD Millipore glucagon radioimmunoassay kit (Merck, Billerica, MA, USA).

#### Incretins

Total GLP-1 and GIP analyses were performed by the Immunoassay Core Biomarker Laboratory, University of Dundee, utilizing MSD metabolic assay total GLP-1 and GIP assay kits.

#### Gliclazide

Gliclazide analysis was performed by the Biomarker and Drug Analysis Core Facility, University of Dundee utilizing a uniquely developed gliclazide quantification method in human plasma by liquid chromatography with tandem mass spectrometry, high-performance liquid chromatography separation, and tandem mass spectrometry analysis.

### Data and statistical analyses

#### Area under curve

The incremental area under the curve (AUC) for insulin and C-peptide were derived via the trapezoidal method for each intervention visit.

#### Incretin effect

The incretin effect was defined as the percentage increase in insulin secretion between oral and intravenous glucose. The classical incretin effect for control and intervention visits were calculated utilizing the following formula (1,14,15):

$$\begin{array}{l} \frac{Incretin\;Effect\left(\%\right)=100\times \left(iAUC_{OGTT}iAUC_{IIGI}\right)}{iAUC_{OGTT}} \end{array}$$

### Study outcomes

The primary outcome was the difference in the classical incretin effect between control and low-dose gliclazide treatment. Beta-cell function derived from modeling was evaluated as a secondary outcome (16,17).

### Power

The power for study was determined via a related study design utilizing hyperglycemic clamps and intravenous incretins. The study by Aaboe et al (18) involved hyperglycemic clamps on 12 subjects with an effect size of ~1.05 with 80% power, and a *P*-value < 0.01 to detect a difference of adding sulphonylurea to GIP. In this study, physiological concentrations of endogenous incretins were anticipated, and lower doses of sulphonylurea were used, thus 20 participants were recruited. The participants from phase 1 receiving the gliclazide stimulus resulting in the largest augmentation of the incretin effect (n = 4) were included in the results analysis with phase 2 participants to total 16 for study. Allowing for 2 noncompleters, 14 participants studied provided 80% power with a significance level of 0.05 to detect an increase over the GLP-1 response of 70% or more observed by Aaboe et al. A *P*-value < 0.05 was considered statistically significant.

### Data presentation

The observed difference in incretin effect for both insulin and C-peptide were compared using a Wilcoxon signed-rank test. As the data were not normally distributed, results are presented as median (lower quartile, upper quartile) unless otherwise stated: results for total GLP-1, GIP, and glucagon were also compared and presented in the same manner. Data for glucose and gliclazide, being normally distributed, were analyzed utilizing paired *t*-test; mean values were calculated as AUC over time during the OGTT, and results are presented as mean ± SEM.

### Modeling

The incretin effect was also assessed using a previously described model, derived from (17,19) and designed to analyze the OGTT and IIGI test simultaneously. The model postulates that during the OGTT incretins potentiate insulin secretion by stimulating early insulin release and enhancing the beta-cell dose-response relationship, which relates insulin secretion to the concomitant glucose concentration. The main model parameters are glucose sensitivity (ie, the mean slope of beta-cell dose-response curve during IIGI); rate sensitivity (representing early insulin release (20,21) from IIGI and from OGTT); glucose-induced potentiation, representing a time-dependent modulation of dose-response during IIGI; and incretin potentiation, quantifying the time course of the incretin effect. The mean incretin effect during the whole OGTT or in the late period was calculated as AUC/time of incretin potentiation. Insulin secretion (pmol min^−1^ m^−2^) was also calculated, as well as its AUC over IIGI or OGTT (nmol/m^2^). Data are presented as mean ± SEM.

### Testing and software

Statistical analyses were conducted through GraphPad Prism 8 for Windows (Version 8.3.1) and R Studio (Version 1.2.1335).

## Results

### Participant characteristics

Study recruitment ran from July 2018 to April 2019. In total, 24 participants were screened for study. Four exclusions were made due to anemia (n = 1), suboptimal HbA1c (n = 2), and comorbidity as deemed by investigators (n = 1). All participants recruited (n = 20) gave informed written consent and completed the study. The baseline characteristics of the study participants are provided in Table 1. The most common concomitant medication included statins (n = 12), anti-hypertensives (n = 11), and proton-pump inhibitors (n = 6).

**Table 1.** Baseline characteristics of study participants by phase of study

Phase of study	Number of participants	Treatment (diet/ metformin)	Sex (M/F)	Age (years)	BMI (kg/m^2^)	HbA1c (mmol/mol)	Duration of diabetes (years)	Age at diagnosis (years)
Full study	20	8/12	10/10	69.5 (9.25)	32.0 (7.8)	50.9 (19)	8 (5.5)	60.5 (7.7)
Phase 1 10 mg	4	2/2	2/2	66.0 (14)	39.7 (7.8)	54.0 (12.3)	10.5 (5.5)	54.5 (10)
Phase 2 20 mg	4	2/2	2/2	70.0 (13)	37.9 (10.2)	45.5 (8.5)	9.0 (6.5)	60.5 (6.3)
Phase 2	16	6/10	8/8	69.5 (10)	32.0 (5.4)	50.0 (7)	8.0 (5.5)	61.0 (5.5)

Data are presented as median (interquartile range).

### Determination of dose

In the exploratory dose-ranging phase, mean glucose was reduced in all participants following low-dose gliclazide intervention, without incidence of hypoglycemia; however, this effect was greater in the 20-mg cohort (mean ± SEM: control 11.3 ± 1.6 mmol/L, gliclazide 10.13 ± 1.4 mmol/L; *P* = 0.018) *vs* the 10-mg cohort (mean ± SEM: control 11.65 ± 1.2 mmol/L, gliclazide 11.03 ± 1.5 mmol/L; *P* = 0.36). Therefore, the 20-mg dose was chosen for the phase 2. In this small cohort, neither dose exhibited augmentation of the classical incretin effect of insulin or C-peptide (Table 2).

**Table 2.** Summary of phase 1 results by dose

	10 mg gliclazide (n = 4)			20 mg gliclazide (n = 4)		
	Control	Gliclazide	*P*-value	Control	Gliclazide	*P*-value
Mean glucose from AUC (mmol/L)	11.7 ± 1.19	11.03 ± 1.47	0.36	11.3 ± 1.64	10.13 ± 1.45	0.02
Incretin effect insulin (%)	24.5 (7.0, 51.5)	27 (21.0, 32.3)	>0.9	31.2 (14.4, 54.5)	56.0 (39.9, 72.8)	0.13
Incretin effect C-peptide (%)	11.3 (-10.6, 36.5)	18.3 (13.5, 20.4)	0.88	27.4 (15.0, 43.9)	23.7 (14.5, 48.3)	0.88
AUC glucagon (oral) (nmol l-1 min)	5.6 (5.1, 7.4)	5.5 (5.1, 7.0)	0.25	5.7 (4.7, 7.3)	6.0 (4.7, 7.8)	0.63
AUC glucagon (IV) (nmol l-1 min)	4.0 (3.5, 5.5)	4.1 (3.3, 5.8)	0.63	4.6 (4.4, 6.1)	4.5 (4.3, 9.8)	0.88
AUC total GLP-1 (nmol l-1 min)	1.1 (0.8, 1.5)	1.1 (0.8, 1.4)	0.88	0.7 (0.6, 1.4)	0.9 (0.7, 2.1)	0.25
AUC total GIP (nmol l-1 min)	13.1 (11.6, 14.8)	14.8 (12.3, 15.3)	> 0.9	10.5 (5.3, 20.1)	9.4 (5.7, 24.4)	0.88

Glucose data presented as mean ± SEM. All other parameters are presented as median (lower quartile, upper quartile).

### Glycemic response

**Figure 2.**
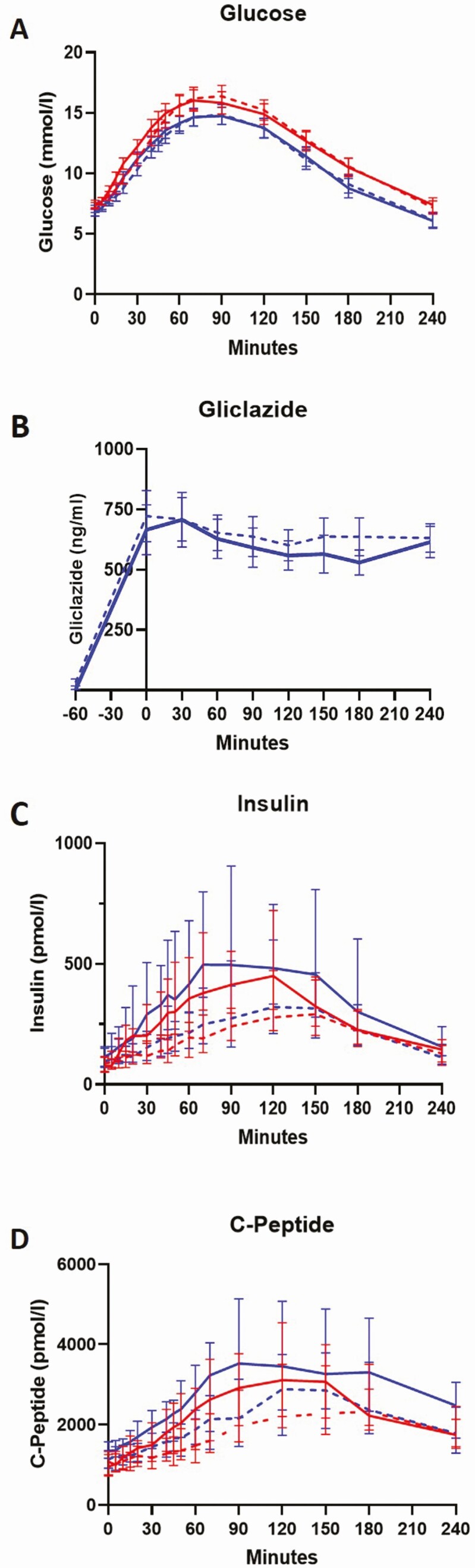
Mean (SEM) plasma glucose (A) and gliclazide (B) and median (interquartile range) insulin (C) and C-peptide (D) concentrations during OGTT (solid lines) and IIGI (dashed lines) in control (red lines) and gliclazide intervention visits (blue lines) in phase 2 (n = 16). For interventions involving gliclazide (B), low-dose gliclazide suspension was administered 60 min prior to the start of the OGTT and IIGI.

Twenty milligrams of gliclazide given 1 h before the OGTT lowered mean glucose level during the OGTT (mean ± SEM: control 12.01 ± 0.56 mmol/L, gliclazide 10.82 ± 0.5 mmol/L; *P* = 0.0006) (Table 3) as well as the mean basal glucose (mean ± SEM: control 7.3 ± 0.28 mmol/L, gliclazide 6.8 ± 0.291 mmol/L; *P* = 0.004). The OGTT-IIGI were well matched (Fig. 2A).

**Table 3.** Summary of incretin effect results from phase 2 (n = 16)

Parameter	Control	Gliclazide	*P*-value
Mean Glucose from AUC (mmol/l)	12.0 ± 0.56	10.8 ± 0.5	<0.01
Incretin Effect Insulin (%)	35.5 (27.3, 61.2)	55.0 (34.8, 72.8)	<0.05
Incretin Effect C-Peptide (%)	28.4 (12.9, 47.0)	39.9 (17.9, 52.8)	<0.05

Data for mean glucose from AUC are given as mean ± SEM. Data for incretin effect for insulin and C-peptide are given as median (lower quartile, upper quartile).

### Insulin, C-peptide, and the incretin effect

**Figure 3.**
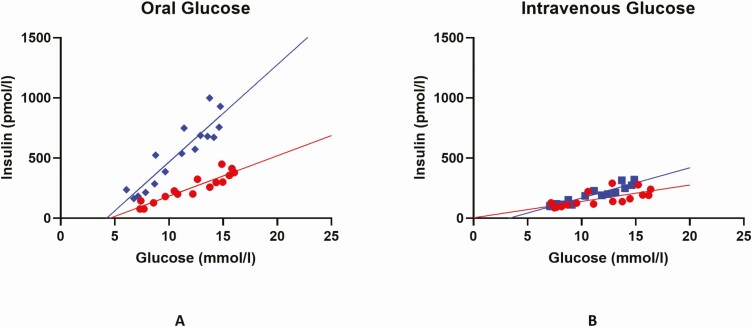
Linear regression analysis of mean insulin secretion against mean plasma glucose levels from phase 2 (n = 16) shows insulin secretion to be significantly augmented in response to oral glucose load (A) compared to intravenous glucose (B) following the addition of low-dose gliclazide (blue lines) *vs* control (red lines). (A) Slope increased from 33.6 to 80.97 (*P* < 0.0001), and (B) slope increased from 13.63 to 25.2 (*P* < 0.01).

Insulin and C-peptide profiles are presented in Fig. 2C and 2D. Table 3 summarizes incretin effect measures derived from AUC values. AUC values are provided in Table 4. The classical incretin effect estimates, as percentages, were increased by 20 mg gliclazide compared to control [median (LQ, UQ)]: incretin effect-_INSULIN_ [control 35.5 (27.3, 62.1), gliclazide 54.99 (34.8, 72.8); *P* = 0.049], incretin effect_c-peptide_ [control 28.4 (12.9, 47.0), gliclazide 39.9 (17.9, 52.8); *P* = 0.049]. When the mean glucose of each participant was regressed against mean insulin, with and without gliclazide, significant augmentation was shown in the slope of insulin secretion in response to oral glucose following gliclazide treatment (control 33.6 *vs* gliclazide 80.97; *P* < 0.001) (Fig. 3A). The slope also increased, but to lesser extent, in the intravenous visits (control 13.63, gliclazide 25.2; *P* = 0.03) (Fig. 3B). There was no difference in incretin effect dependent on baseline gender, HbA1c, pre-existing diabetes treatment, or duration of diabetes.

**Table 4.** Summary of AUC results from phase 2 (n = 16)

Parameter	Control	Gliclazide	*P*-value
AUC insulin (nmol l^−1^min^−1^)			
OGTT	68.8 (48.5)	91.2 (84.9)	0.01
IIGI	51.7 (29.7)	55.5 (37.2)	0.38
AUC C-peptide (nmol l^−1^min^−1^)			
OGTT	566 (306)	609 (362)	<0.01
IIGI	448 (362)	528 (306)	0.19
AUC total GLP-1 (nmol l^−1^min^−1^) OGTT	1.04 (0.9, 1.5)	1.0 (0.7, 1.8)	0.25
AUC total GIP (nmol l^−1^min^−1^) OGTT	15.1 (9.1, 19.3)	13.6 (11.6, 19.9)	0.51
AUC glucagon (nmol l^−1^min^−1^)			
OGTT	4.9 (4.5, 5.6)	4.6 (4.4, 5.8)	0.56
IIGI	3.8 (3.3, 4.7)	3.8 (3.4, 4.5)	0.63

Results for insulin, C-peptide, and glucagon (OGTT and IIGI) and total GLP-1 and total GIP (OGTT only) are given as median (lower quartile, upper quartile).

### Modeling of beta-cell function and the incretin effect

**Figure 4.**
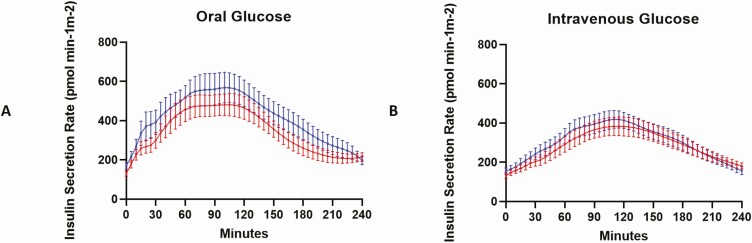
Incretin secretion profiles 0 to 240 min in response to (A) oral glucose and (B) intravenous glucose (mean ± SEM) in phase 2 (n = 16).

**Figure 5.**
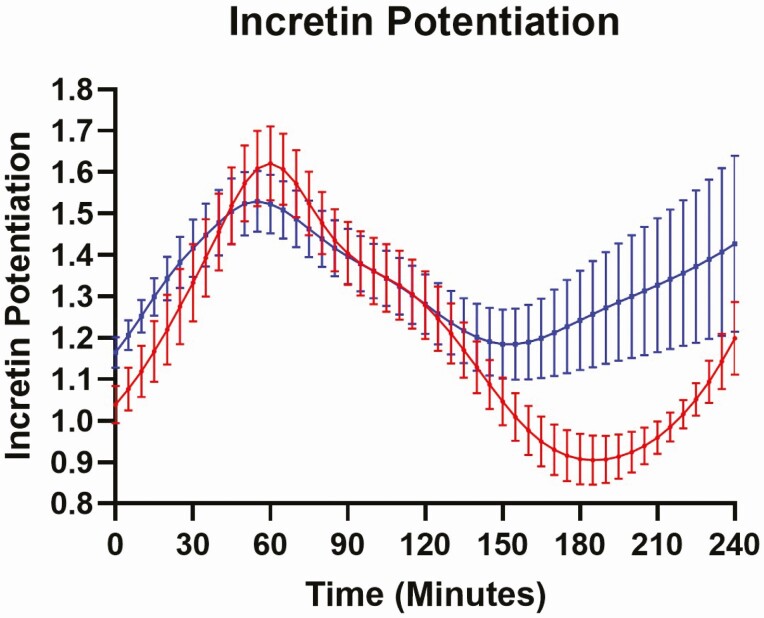
Incretin potentiation profiles from 0 to 240 min for control (red) and gliclazide intervention (blue) in phase 2 (n = 16).

Insulin secretion rates for OGTT and IIGI in the 2 treatment groups were similar, with an apparent sustained incretin effect in late stages of OGTT (Fig. 4). The beta-cell function parameters derived from modeling of beta-cell function are shown in Table 5. Beta-cell glucose sensitivity (mean ± SEM; calculated from IIGI) increased from 22.61 ± 3.94 in the control to 33.11 ± 7.83 (*P* = 0.01) by gliclazide. The incretin potentiation factor overall was no different between control and gliclazide treatment, but there was an increase in late-phase incretin potentiation with an increase in AUC/time from 180 to 210 min (Fig. 5).

**Table 5.** Summary of results from beta-cell modeling (mean ± SEM) from phase 2 (n = 16)

	Control	Gliclazide	*P*-value
Glucose sensitivity (pmol min^-1^m^-2^l mmol^-1^)	22.6 ± 3.94	33.1 ± 7.83	0.01
Rate sensitivity (pmol m^−2^ lmmol^−1^)			
OGTT	265 ± 51.2	370 ± 137	>0.9
IIGI	181 ± 51.2	119 ± 58.4	0.09
Incretin potentiation integral mean (AUC/time)			
0-240 min	1.2 ± 0.05	1.3 ± 0.08	0.35
180-210 min	0.92 ± 0.05	1.29 ± 0.14	0.04
Total insulin secretion rate (nmol/min^−2^)			
OGTT	81.8 ± 9.1	99.7 ± 13.0	<0.01
IIGI	68.3 ± 7.9	72.9 ± 7.6	0.27

### Glucagon, GLP-1, and GIP

Glucagon secretion was unaffected by the presence of gliclazide in OGTT [mean ± SEM: AUC_GLUCAGON_ control 4.9 (4.5, 5.6) nmol l^−1^min^−1^, gliclazide 4.9 (4.4, 5.8) nmol l^−1^min^−1^; *P* = 0.56]. There was no impact of gliclazide on the time course of total GLP-1 or GIP as well as AUC. AUC_TotalGLP-1_ [mean ± SEM: control 1.04 (0.9, 1.5) nmol l^−1^min^−1^, gliclazide 1.0 (0.7, 1.8) nmol l^−1^min^−1^; *P* = 0.2524] and AUC_TOTALGIP_ (mean ± SEM: control 15.06 (9.1, 19.3) nmol l^−1^min^−1^, gliclazide 13.6 (11.6, 19.9) nmol l^−1^min^−1^; *P* = 0.51] (Table 4).

### Gliclazide pharmacokinetics

The time course of gliclazide concentration is shown in Figure 2B. The mean gliclazide concentrations from AUC were 681.2 ± 83.5 ng/mL for OGTT and 742.2 ± 85.5 ng/mL for IIGI (*P* = 0.12), respectively. Some difference was noted in gliclazide concentrations between oral and intravenous tests between 120 and 240 min during which mean gliclazide concentrations were 563.5 ± 58.3 ng/mL and 625.4 ± 67.1 ng/mL (*P* = 0.06) for OGTT and IIGI, respectively.

## Discussion

First, in a cohort of White British patients with type 2 diabetes on diet or metformin monotherapy, we have shown that low-dose gliclazide, resulting in drug concentrations far below those seen with normal “therapeutic” doses, significantly reduces plasma glucose. Although low doses of gliclazide can be used successfully in patients with hepatic nuclear factor 1α maturity-onset diabetes of the young due to extreme sulphonylurea sensitivity (22), only 1 other Japanese study in adult type 2 diabetes has been conducted that showed improvements in glycemic control with low-dose gliclazide (23). However, this study did not assess acute physiological response to glucose load; in addition, although this study reported increased rates of hypoglycemia in the gliclazide cohort, this outcome was only assessed on symptom reporting without recording of blood glucose.

Second, for the first time using low-dose gliclazide, we show that sulphonylureas augment the classical incretin effect in patients with type 2 diabetes, in addition to the known effects on glucose and insulin secretion. This mechanism has been suggested previously using therapeutic doses of glipizide and GIP infusion in hyperglycemic clamps; Aaboe et al showed that when 10 mg glipizide was administered prior to GIP infusion there was potential synergy between sulphonylurea and GIP (18). In our study, we used low-dose gliclazide and show augmentation of endogenous incretin response in type 2 diabetes, suggesting a potential therapeutic use and novel mechanism for low-dose sulphonylureas in patients with type 2 diabetes. This is supported as the slope of insulin secretion dependent on glucose was augmented in response to gliclazide intervention following oral glucose but not intravenous glucose (Fig. 3). Furthermore, trendline prediction shows them to cross around the level of 5, suggesting that sulphonylureas at a low dose are unlikely to stimulate insulin secretion below this threshold. This is in contrast to high-dose sulphonylureas, which have been shown to dissociate the glucose dependency of GLP-1 (24). We show no impact of low-dose gliclazide on secretion of GLP-1, GIP, or glucagon, all supporting a role for the triggering pathway to augment incretin action.

We hypothesized that low-dose sulphonylureas may work to increase incretin action with only minimal activation of the triggering pathway and, similar to the mechanism for glucose responsiveness in K_ATP_-neonatal diabetes mellitus treated with sulphonylurea, conjectured that this would be due to a partial closure of K_ATP_ channels resulting in a rise in beta-cell membrane potential to a level where the incretin pathway may act. Although we have established that low-dose gliclazide does indeed increase the incretin effect, the potential mechanisms that underlie such an effect remain unclear. We suggest a complex interaction of mechanisms, which, in addition to membrane electrophysiology (25), may also include a role for intracellular calcium, which has been shown to control exocytosis by mediating emptying of immediately releasable pools (26). This is supported by the fact that low-dose sulphonylurea increased glucose sensitivity, a measure of the amplifying pathway, by 50% in response to intravenous glucose (ie, in the absence of increased incretins) (26).

An additional alternative mechanism from our analysis suggests that the increase in incretin effect observed with gliclazide treatment might be mediated by improvement of basal glucose as some studies suggest that glucose lowering partially restores the action of incretins (27), with heightened insulin response to supraphysiological GIP or GLP-1 infusion noted following 4 weeks of intensive glucose lowering (28). However, in our study, the gliclazide is only given 1 h before the glucose stimulus so this mechanism seems unlikely.

This is the first study to report pharmacokinetic parameters of 20 mg gliclazide suspension. A single dose of 20 mg gliclazide suspension achieved C_MAX_ of ~700 ng/mL (Fig. 2B). This concentration following a single acute dose of gliclazide remained steady for the 4-h intervention period in both oral and intravenous studies. For comparison, a single dose of 40 to 120 mg immediate release gliclazide in tablet formulation achieves C_MAX_ of 2200 and 8000 ng/mL, respectively, whereas 30 mg modified release gliclazide achieves C_MAX_ ~800 ng/mL with a half-life of 15 h (29,30). Thus, the 20 mg dose given 1 h before the isoglycemic clamp study resulted in gliclazide concentrations far lower than seen with normal therapeutic doses of the immediate release tablet formulations of gliclazide. It is interesting to note that the modified release formulation of gliclazide results in gliclazide concentrations more like those seen in our study. A 30-mg dose of modified release gliclazide, albeit with differing pharmacokinetic profile, is as effective at glucose lowering as 80 mg of immediate release gliclazide (30); our study suggests that some of the glycemic benefit of exposure to lower concentrations of gliclazide may be mediated by augmentation of incretin action. Consistent with this, modified release sulphonylureas are associated with lower risk of hypoglycemia when compared with standard release preparations such as glibenclamide/glyburide (31) and glimepiride (32) and result in lower weight gain and slower time to failure compared with glibenclamide (33) and chlorpropamide (34). Further pharmacokinetic studies of low-dose gliclazide are ongoing.

This study has strengths in the use of the gold-standard technique for physiological evaluation of the incretin effect and demonstrates results in line with observations from previous incretin physiology studies in type 2 diabetes (1,35). The main limitation of study is the wide variability in beta-cell response within a small sample size, which limits power and the ability to perform subanalyses; for example, the study is underpowered to fully evaluate trends in phenotypes of responders. Furthermore, this study recruited only well-controlled type 2 diabetics; however, no difference was noted in response dependent on HbA1c or duration of diabetes. The study design utilized open-label gliclazide, with modest glucose-lowering effect; therefore, a formal randomized controlled trial would be required to further evaluate these outcomes. Furthermore, the authors acknowledge the use of acute, not chronic, gliclazide dosing in this physiological study. The advantage of beta-cell modeling over classical incretin effect indices is the ability to model the time course of the incretin effect. It also accounts for possible small differences in glucose between OGTT and IIGI tests. However, as a more complicated procedure and by providing multiple parameters, it may add some estimation error. This would explain why significance is obtained with the classical indices but not with the model incretin potentiation AUC.

## Conclusion

In this study we have shown that low-dose gliclazide reduces plasma glucose in response to oral glucose load, with concomitant augmentation of the classical incretin effect assessed by insulin and C-peptide secretion. In addition, modeling of beta-cell function shows that low plasma concentrations of gliclazide particularly potentiate late-phase insulin secretion and increase glucose sensitivity by 50%. We propose that low-dose sulphonylureas, with doses comparable, or lower than those seen with gliclazide modified release 30 mg, may result in effective glucose lowering with reduced risk of hypoglycemia. Given the low cost of sulphonylureas, trials of low-dose sulphonylureas in low- and middle-income countries are warranted.

## Data Availability

Some or all data sets generated and/or analyzed during the current study are not publicly available but are available from the corresponding author on reasonable request.

## Notes

***Financial Support:*** This research was funded by a New Investigator Award to ERP from Wellcome (102820/Z/13/Z).

***Clinical Trial Information:*** ClinicalTrials.gov: NCT03705195.

***Author Contributions:*** RLMC and ERP conceived, designed, and acquired study data. RLMC undertook statistical analysis with advice from ERP and AM. AM and AT conducted beta-cell modeling analysis and interpretation. RLMC and EP wrote the initial drafts of the manuscript; these drafts were revised for important scientific content by all authors. All authors gave final approval of the version to be published. ERP is the guarantor of this work.

## Additional Information

***Disclosures:*** RLMC has been awarded the Rising Star Educational Program Co-Sponsored by the International Diabetes Center and Sanofi. AM has received research grants from Boehringer Ingelheim and Lilly and consultancy fees from Lilly. ERP has received honoraria for speaking from Lilly and has received public-private partnership research funding as part of the EU Framework 7 and Horizon 2020 Innovative Medicines Initiative from Boehringer-Ingelheim, Johnson and Johnson, Lilly, Novo Nordisk, Sanofi, and Servier.
